# Bidirectional regulation between mitochondrial metabolic reprogramming and epigenetic modifications in renal tubular epithelial cell injury of diabetic kidney disease

**DOI:** 10.3389/fendo.2026.1831426

**Published:** 2026-07-30

**Authors:** Guijun Wu, Fanghong Chen, Qing Xiong

**Affiliations:** 1 Department of Endocrinology, The Affiliated Lianyungang Oriental Hospital of Kangda College of Nanjing Medical University/Lianyungang Municipal Oriental Hospital, Lianyungang, Jiangsu, China; 2 Department of Endocrinology, The Affiliated Haikou Hospital of Xiangya Medical College, Central South University, Haikou, Hainan, China

**Keywords:** bidirectional crosstalk, diabetic kidney disease (DKD), epigenetic modification, metabolic memory, mitochondrial metabolic reprogramming, renal tubular epithelial cell (RTEC) injury

## Abstract

**Background:**

Diabetic kidney disease (DKD) is the leading cause of end-stage renal disease (ESRD) worldwide. Renal tubular epithelial cell (RTEC) injury is a core driver of DKD initiation and progression. Mitochondrial metabolic reprogramming and epigenetic modification are two core events in DKD pathogenesis, and their bidirectional crosstalk has become a frontier and hot research topic in the pathogenesis of DKD. At present, the specific molecular mechanisms of their bidirectional regulation remain incompletely elucidated.

**Methods:**

This narrative review collected literatures from PubMed, Web of Science and Embase up to March 2026. English original articles and reviews were included, whereas case reports, letters and non-English publications were excluded. We summarized the latest advances concerning the interaction between mitochondrial metabolic reprogramming and epigenetic modification in RTEC injury of DKD, focusing on their bidirectional molecular regulation.

**Results:**

Key mitochondrial metabolic intermediates (acetyl-CoA, α-ketoglutarate (α-KG), nicotinamide adenine dinucleotide (NAD^+^)) act as substrates or cofactors of epigenetic enzymes to regulate DNA methylation, histone modifications, and noncoding RNA expression in RTECs. Epigenetic modifications in turn remodel mitochondrial biogenesis, fatty acid oxidation, and oxidative phosphorylation by regulating the expression of metabolism-related genes.

**Conclusion:**

This narrative review elaborates the closed-loop crosstalk between mitochondrial metabolism and epigenetics in tubular injury, highlights the therapeutic prospect of targeting this bidirectional regulatory axis, and provides novel theoretical evidence for revealing DKD pathogenesis and developing targeted intervention regimens.

## 1 Introduction

Diabetic kidney disease (DKD) is a major microvascular complication of diabetes mellitus, and has become the leading cause of chronic kidney disease (CKD) and end-stage renal disease (ESRD) worldwide. Approximately 40% of individuals with diabetes develop DKD during their lifetime (1). The pathogenesis of DKD involves multiple factors, including metabolic disorders, oxidative stress, inflammation, and fibrosis. DKD has long been regarded as a predominantly glomerular disease, and earlier studies have focused primarily on glomerular basement membrane thickening, mesangial matrix expansion, and podocyte injury and glomerulosclerosis. However, renal tubular epithelial cells (RTEC) are the main functional cells of the kidney, responsible for reabsorption and secretion, and their dysfunction directly leads to renal function decline. Recent studies in recent years have shown that RTECs exhibit dysfunction in early-stage DKD, and tubular injury correlates more strongly with renal function decline than glomerulopathy does, with a correlation coefficient of r = 0.67 (2).

Mitochondria, the cellular “powerhouse”, are particularly abundant in RTECs to sustain their high-capacity reabsorptive function. In DKD, metabolic stress such as high glucose and high fat induces mitochondrial dysfunction, which in turn elicits adaptive metabolic alterations, a process termed “mitochondrial metabolic reprogramming” (3). Metabolic reprogramming is characterized by impaired oxidative phosphorylation (OXPHOS), enhanced glycolysis, impaired fatty acid oxidation (FAO), and abnormal lipid accumulation in RTECs, ultimately leading to oxidative stress, inflammation, and apoptosis (4).

At the same time, the role of epigenetic modifications in the pathogenesis of DKD has attracted increasing attention. DNA methylation, histone modification, and noncoding RNA regulation can persistently affect gene expression by altering chromatin structure and gene accessibility without changing the DNA sequence (5). Notably, metabolic reprogramming and epigenetic modification are not two isolated pathological processes but form a complex regulatory network. Metabolic intermediates are directly involved in the activity regulation of epigenetic modifying enzymes, while epigenetic mechanisms affect metabolic status by regulating the expression feedback of metabolic genes, forming a complex bidirectional regulatory network (6, 7).

However, the current reviews on DKD focus mostly on a single mechanism: either mitochondrial metabolic reprogramming, or epigenetic modification (3, 8). Few reviews have summarized the bidirectional crosstalk between them and their specific regulatory role in RTEC injury of DKD. Notably, most available evidence centers on proximal tubular epithelial cells, while cell-type-specific mechanisms in distal tubules, collecting duct cells, glomerular-tubular crosstalk, and immune cell contributions remain poorly defined. Addressing this heterogeneity is critical for translating basic findings into precise therapies. This review integrates the latest research evidence to propose a theoretical framework of bidirectional regulation, which fills the gap of existing reviews focusing on a single mechanism. This review first elaborates on the roles of mitochondrial metabolic reprogramming and epigenetic modification in RTEC injury, then focuses on their bidirectional regulatory mechanisms, and finally discusses the therapeutic prospects, providing a theoretical basis for DKD treatment.

To overcome these drawbacks, this review moves beyond simple parallel description of independent pathways via a hypothesis-oriented analytical perspective. We explicitly distinguish between upstream triggers and secondary adaptive responses, differentiate early maladaptive metabolic shifts from late-stage fibrotic events, and highlight cell-type-specific heterogeneity along the nephron. Moreover, we discuss unresolved controversies, including the lack of direct *in vivo* evidence for certain epigenetic-metabolic loops in renal tubules, and the translational limitations of current therapeutic strategies. This framework aims to provide a conceptual hierarchy specific to DKD rather than extrapolating generalized paradigms from cancer or immunometabolism.

This study is a narrative review focusing on the bidirectional crosstalk between mitochondrial metabolic reprogramming and epigenetic modifications in RTEC injury of DKD. This narrative review synthesizes current evidence without formal systematic literature search or meta-analytic methods. We conducted a literature search across PubMed, Web of Science, and Embase through March 2026, using keywords including “diabetic kidney disease”, “renal tubular epithelial cell injury”, “mitochondrial metabolic reprogramming”, “epigenetic modifications” and “bidirectional regulation”. Original articles, reviews, and preclinical studies published in English were included, while letters, case reports, and non-English publications were excluded. Studies were selected based on their relevance to the molecular mechanisms linking metabolism and epigenetics in DKD-related tubular injury. No formal systematic review methodology, such as risk-of-bias assessment or quantitative synthesis, was performed.

**Figure 1:**
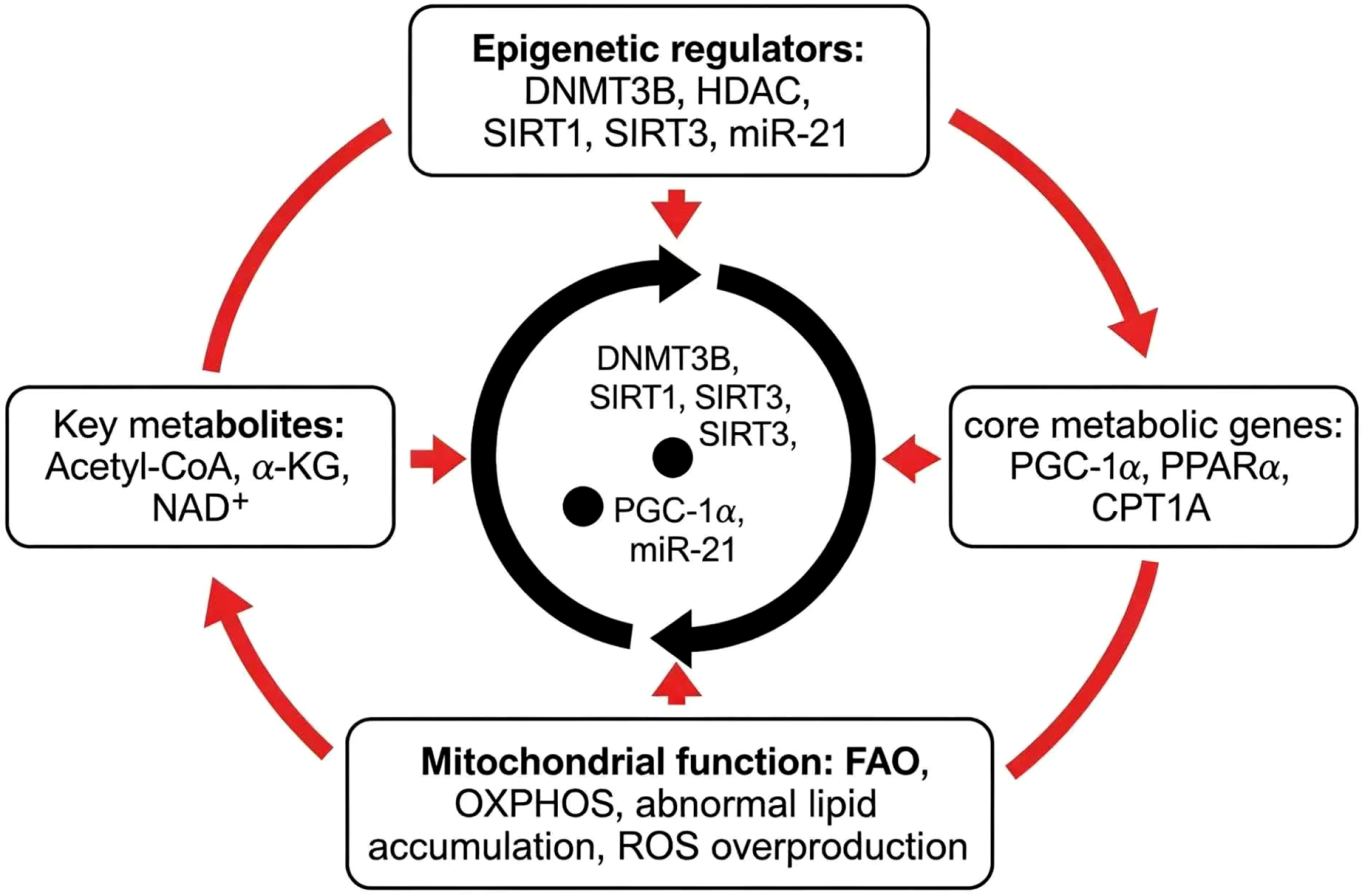
Schematic diagram of closed-loop bidirectional regulation between mitochondrial metabolites and epigenetic modification in RTEC injury of DKD.

Based on the systematically sorted existing preclinical and clinical evidence, we originally propose a closed-loop bidirectional regulatory hypothesis focused on RTEC injury in DKD: mitochondrial metabolic intermediates modulate epigenetic enzyme activity to alter DNA methylation, histone modification and non-coding RNA expression; the resultant epigenetic remodeling transcriptionally represses core metabolic genes including PGC-1α, PPARα, and CPT1A, further impairing mitochondrial biogenesis, FAO and oxidative phosphorylation; dysfunctional mitochondria in turn change the intracellular pool of acetyl-CoA, α-KG and NAD^+^ to feedback regulate epigenetic status, finally forming a persistent pathological closed circuit dominated by core nodes DNMT3B (DNA methyltransferases 3B), SIRT3 and PGC-1α. To visualize this hypothesis, we construct an integrated bidirectional regulatory loop model diagram (**Figure 1**) to illustrate the complete cascade of “metabolites → epigenetic modifications → metabolic-related genes → mitochondrial function → endogenous metabolites”. This self-amplifying pathological loop provides a novel theoretical interpretation for the initiation and persistence of tubular damage as well as DKD-related metabolic memory.

## 2 Role of mitochondrial metabolic reprogramming in renal tubular injury in DKD

### 2.1 Concept and connotation of mitochondrial metabolic reprogramming

Metabolic reprogramming refers to the adaptive shift in cellular metabolic programs to meet energy demands and adapt to environmental stress (9). In the context of DKD, RTECs show significant metabolic remodeling, which is characterized by impaired mitochondrial biogenesis and impaired oxidative phosphorylation, accompanied by compensatory enhancement of glycolysis, as well as impaired FAO and dysregulated lipid metabolism (4).

The kidney is a high-energy-consuming organ second only to the heart (10). RTECs are rich in mitochondria and mainly rely on FAO for 70%—75% of ATP production (11). Under physiological conditions, fatty acids enter the mitochondrial matrix through carnitine palmitoyltransferase 1 (CPT1) and undergo β-oxidation to generate acetyl-CoA, which enters the tricarboxylic acid cycle (TCA cycle) to generate reducing equivalents of NADH and FADH2, and finally drives oxidative phosphorylation through the electron transport chain (12). This highly efficient energy metabolic network maintains renal tubular reabsorption function and cellular homeostasis.

### 2.2 Characteristics of renal tubular mitochondrial metabolic reprogramming in DKD

In the DKD state, high glucose environment induces the overproduction of mtROS (mitochondrial reactive oxygen species) by activating the nicotinamide adenine dinucleotide phosphate (NADPH) oxidase pathway, which impairs the activity of mitochondrial DNA and respiratory chain complexes, leading to reduced efficiency of oxidative phosphorylation (13). To maintain energy supply, cells undergo an adaptive metabolic switch: glycolytic flux increases, pyruvate entering mitochondria decreases, leading to lactate accumulation, which further aggravates RTEC dysfunction and renal interstitial acidosis; At the same time, the expression of key enzymes of FAO is down regulated, and fatty acid utilization is impaired, resulting in abnormal lipid accumulation in the cytosol (14).

Studies have shown that the expression of key FAO enzymes, carnitine palmitoyltransferase 1A (CPT1A), acetyl-CoA carboxylase (ACC) and peroxisome proliferator activated receptor alpha (PPARα), is significantly decreased in kidney tissues of DKD patients and animal models, detected by Western blot and real-time quantitative PCR (qRT-PCR) (15). The impairment of FAO not only reduces ATP production, but also leads to the accumulation of lipid intermediates such as long-chain acylcarnitines and triglycerides, triggering lipotoxicity and cellular dysfunction. These lipid molecules can activate endoplasmic reticulum stress and inflammatory signaling pathways, and promote renal tubular epithelial cell apoptosis and interstitial fibrosis.

Hou et al. (16) found that mitochondrial oxidative damage itself can drive lipid metabolism reprogramming. Using mitochondria-targeted antioxidant peptide SS31 to protect mitochondrial cristae structure can significantly reverse the down-regulation of FAO key enzymes in DKD RTECs and reduce lipid peroxidation and sphingolipid deposition (16). This finding suggests that mitochondrial dysfunction is not only the result of metabolic reprogramming, but also its upstream initiator.

### 2.3 Regulatory mechanisms of mitochondrial metabolic reprogramming

Mitochondrial metabolic reprogramming is tightly regulated by multiple signaling and transcriptional networks. As a cellular energy sensor, AMP-activated protein kinase (AMPK) is activated when energy is scarce and promotes mitochondrial biogenesis and FAO by phosphorylating its downstream targets such as PGC-1α, PPARα and CPT1A to promote mitochondrial biogenesis and FAO (13). peroxisome proliferator-activated receptor γ coactivator 1α (PGC-1α) is a core regulator of mitochondrial biogenesis, and its expression is significantly downregulated in DKD renal tubules, which is closely related to mitochondrial dysfunction (16).

In addition, hypoxia-inducible factor 1α (HIF-1α) is stably expressed in DKD renal tubules, which can promote the transcription of glycolysis-related genes and exacerbate metabolic switching (17). P53, abnormally upregulated in DKD to inhibit mitochondrial biogenesis and promote apoptosis, together with SIRT1 (downregulated in DKD, promoting mitochondrial biogenesis/FAO via PGC-1α deacetylation) and SIRT3 (reduced activity in DKD, protecting mitochondria by inhibiting ROS (reactive oxygen specie) and activating antioxidant enzymes), participates in the mitochondrial metabolic regulatory network, and their dysregulation contributes critically to metabolic reprogramming in DKD (13).

**Figure 2:**
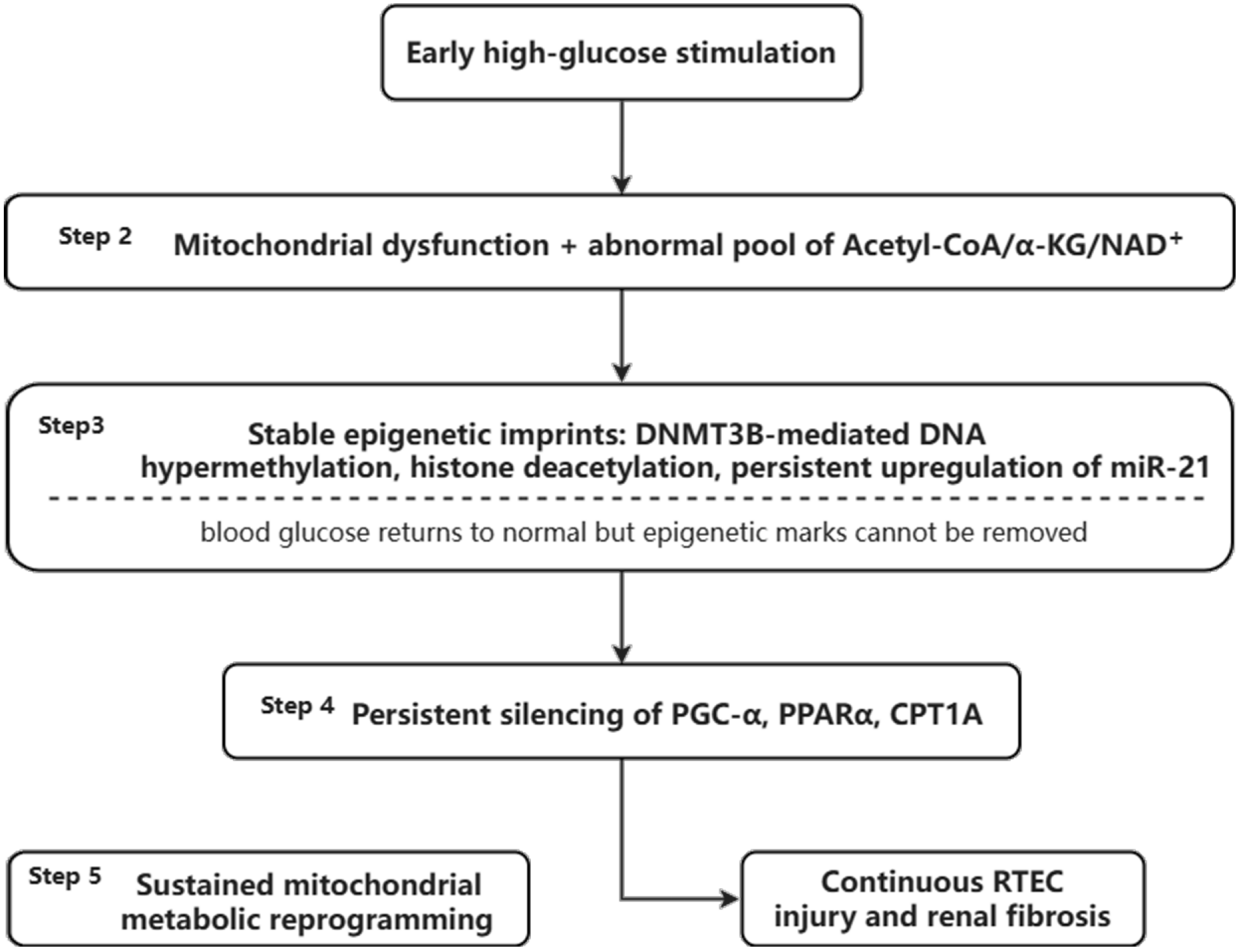
Molecular mechanism of metabolic memory in RTEC of DKD.

Collectively, the multiple upstream signaling cascades converge to drive disordered mitochondrial substrate metabolism and adaptive metabolic shift in DKD RTECs. The core pathological features and regulatory network of tubular mitochondrial metabolic reprogramming are summarized in **Figure 2**.

## 3 Role of epigenetic modification in renal tubular injury in DKD

### 3.1 DNA methylation

DNA methylation refers to the addition of methyl groups to the 5’-carbon atom of cytosine of CpG dinucleotides under the catalysis of DNA methyltransferases (DNMTs) to forming 5-methylcytosine (5-mC). This modification typically induces transcriptional silencing. In DKD renal tubular injury, dysregulated DNA methylation modulates the expression of multiple key genes (18).

Accumulating evidence indicates that DNMT3B is significantly upregulated in DKD RTECs and silences SFRP5 expression by increasing the methylation level of its promoter region (19). Loss of SFRP5 expression leads to abnormal activation of Wnt/β-catenin pathway and promotes extracellular matrix deposition and renal fibrosis.

The use of methyltransferase inhibitor 5-azacytidine (5-AzaC) or knockdown of DNMT3B restores SFRP5 expression and attenuates renal fibrosis (20). This study directly demonstrated the pathogenic role of DNA methylation modification in renal tubular injury in DKD.

In addition, the methylation status of CD36 gene is associated with renal tubular lipid uptake and FAO function. CD36 deletion restores mitochondrial function through the PDK4-AMPK axis and alleviates kidney injury in DKD (21).

### 3.2 Histone modification

Histone modifications (acetylation, methylation, phosphorylation, ubiquitination, etc.) affect gene transcription by changing chromatin structure and recruiting downstream regulatory complexes. Histone acetylation, usually associated with transcriptional activation, is catalyzed by histone acetyltransferases (HATs). In contrast, histone deacetylation is mediated by histone deacetylases (HDACs) and sirtuin (SIRT) family proteins.

In DKD renal tubules, HDAC activity is abnormally elevated, leading to histone deacetylation and gene silencing of a variety of antioxidant and metabolic genes. SIRT1 and SIRT3 are NAD^+^-dependent deacetylases that localize to the nucleus and mitochondria and regulate metabolism and stress responses. In DKD state, the decrease in NAD^+^ levels can reduce the activity of SIRT1/3 deacetylase, leading to highly acetylated inactivation of mitochondrial antioxidant proteins (SOD2/IDH2) (superoxide Dismutase 2/isocitrate Dehydrogenase 2), respiratory chain complexes, and PGC-1α, causing ROS accumulation, decreased respiratory function, and impaired biosynthesis, ultimately resulting in mitochondrial dysfunction and oxidative stress (22).

Histone methylation modifications are also involved in the pathogenesis of DKD. Methyltransferases SET7/9 and demethylase LSD1 regulate the expression of inflammation-and fibrosis-related genes. Recent studies have shown that β-Hydroxybutyric acid is a metabolic intermediate that acts as an endogenous HDAC inhibitor on the nucleus of human renal cortical epithelial cells, protecting the kidneys by increasing histone acetylation levels (23).

### 3.3 Non-coding RNA regulation

miRNAs inhibit autophagy related gene expression by targeting mRNA, lncRNAs regulate autophagy pathways at transcriptional and post transcriptional levels, and circRNAs mainly act as ceRNA (competing endogenous RNA) sponges to adsorb miRNAs. These three factors work together to precisely regulate autophagy processes in kidney disease by directly targeting autophagy genes or regulating signaling pathways such as MTOR/AMPK/PI3K-AKT (24). miRNAs suppress translation or induce mRNA degradation by binding to the 3′UTR of target mRNAs. Several miRNAs (miR-21, miR-192, miR-29) are involved in regulating the metabolism, inflammation, and fibrosis of renal RTECs (25).

LncRNAs can regulate gene expression by sponging miRNAs and recruiting chromatin modification complexes. In DKD, a high glucose environment leads to a decrease in methyltransferase-like 3 (METTL3) expression and a reduction in m6A modification mediated by it, resulting in insulin-like growth factor 2 mRNA-binding protein 2 (IGF2BP2) being unable to stabilize lncRNA TUG1 and downregulating its expression. TUG1 can bind to and activate the PGC-1α promoter to promote its transcription, thereby upregulating Nrf1, Nrf2, and TFAM to increase mtDNA content, ATP levels, and mitochondrial complex I/III activity, reduce ROS generation, and improve mitochondrial morphology, ultimately alleviating renal tubular mitochondrial dysfunction (26). METTL3 downregulates CircRNAs through YTHDF2 mediated m6A methylation, which upregulates ATG4B through sponge adsorption of miR-665-3p, regulating podocyte autophagy, reducing damage and inflammation. Its absence exacerbates DKD progression through this axis (27).

**Figure 3:**
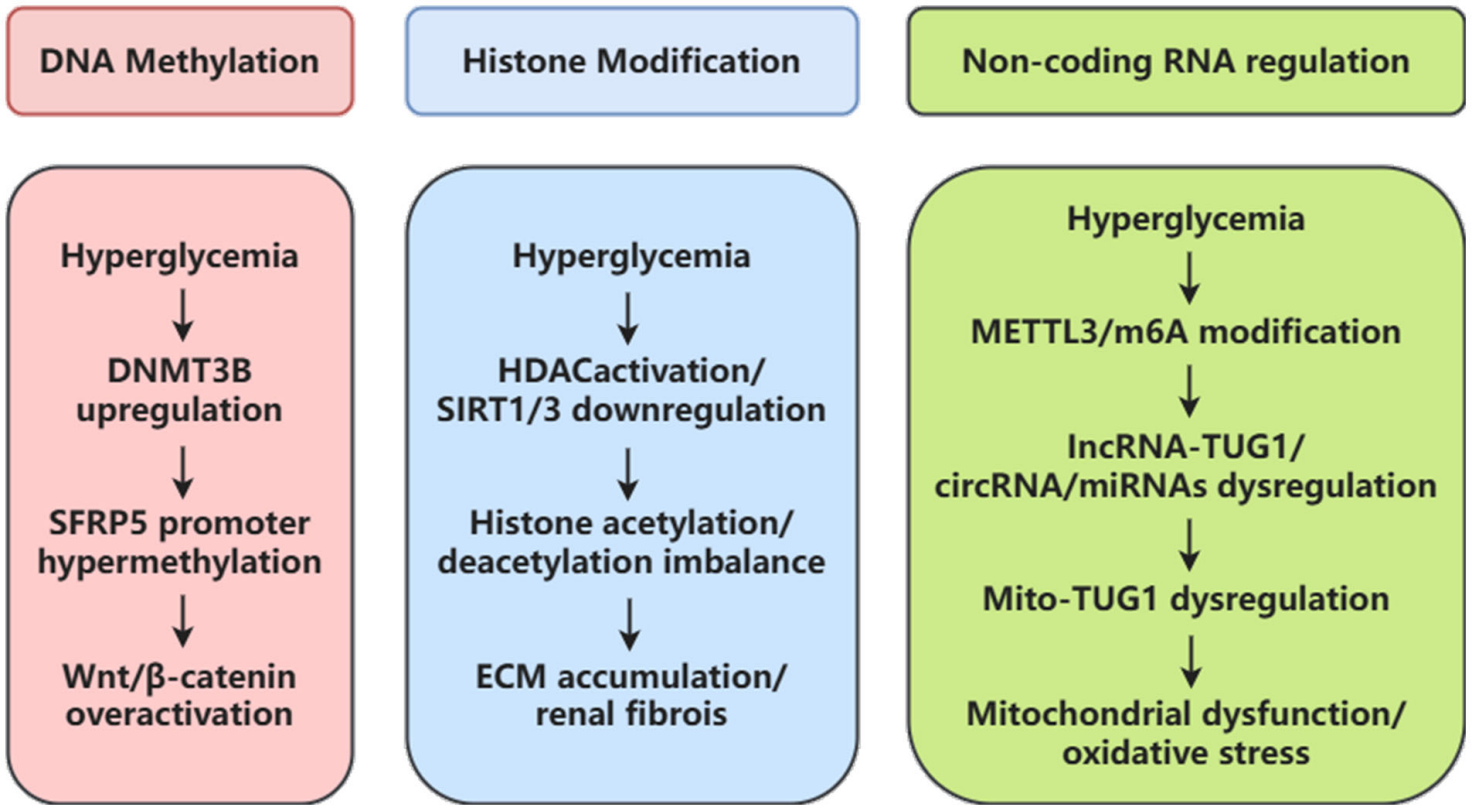
Three major epigenetic regulatory patterns driving RTEC injury in DKD.

As elaborated above, DNA methylation, histone modification and non-coding RNA-mediated epigenetic regulation independently disturb the transcription of key metabolic and fibrosis-related genes in DKD tubular cells. **Figure 3** systematically illustrates the detailed molecular targets and downstream pathological effects of three major epigenetic patterns during RTEC injury.

## 4 Bidirectional regulation of mitochondrial metabolic reprogramming and epigenetic modifications

A critical appraisal of the bidirectional regulation concept is necessary before detailing its molecular basis. While the notion that metabolic intermediates influence epigenetic enzymes and vice versa is well established in cancer and immunometabolism, its application to DKD requires careful reinterpretation. First, many epigenetic-metabolic loops proposed in DKD are inferred from non-renal models and lack direct causal evidence in injured tubular epithelial cells. Second, distinguishing primary drivers from secondary consequences remains challenging: for example, NAD^+^ depletion may be an early event driven by hyperglycemia, whereas DNA hypermethylation of PGC-1α could be a late maladaptive response. Third, adaptive versus maladaptive outcomes depend on disease stage and metabolic context-enhanced glycolysis may initially compensate for mitochondrial dysfunction but eventually exacerbate injury. The following sections synthesize existing evidence with these caveats in mind.

### 4.1 Metabolic intermediates act as substrates and cofactors for epigenetic modifications

Mitochondrial metabolic intermediates are directly involved in the activity regulation of epigenetic modifying enzymes, forming the molecular basis of metabolic reprogramming affecting epigenetics.

Acetyl-CoA, as the core acetyl donor of histone acetylation, directly regulates the catalytic activity of HAT at the intracellular level through substrate accessibility, thereby changing the level of histone acetylation modification, regulating chromatin opening status and gene expression, and achieving the coupling of cellular metabolic status and epigenetic regulation (28). Acetyl-CoA is mainly derived from glucose metabolism (pyruvate dehydrogenase pathway) and fatty acid β-oxidation. In DKD renal tubules, impaired FAO leads to reduced acetyl-CoA production, which may limit histone acetylation levels and affect the expression of metabolic and antioxidant genes (29). As an intermediate product of the TCA cycle, citric acid can be converted into acetyl-CoA by ATP-citrate lyase (ACLY), connecting the TCA cycle and epigenetic regulation.

α-Ketoglutarate (α-KG) is a pivotal TCA cycle intermediate and a cofactor of a variety of dioxygenases, including DNA demethylases (TET family) and histone demethylases (JMJC family). The decrease in α-KG levels renders it unable to serve as an essential cofactor for TET family DNA demethylases and JMJC domain histone demethylases, resulting in inhibited demethylation catalytic activity of both enzymes, leading to DNA and histone hypermethylation and regulation of related gene expression (30). In DKD, mitochondrial dysfunction may lead to reduced α-KG production and promote aberrant methylation. Interestingly, glutaminolysis can supplement the α-KG pool, and this compensatory mechanism may be activated in DKD (31).

NAD^+^ is a critical cofactor for SIRT1–3 deacetylases and PARP enzymes. NAD^+^ level reflects cellular energy status, and its decline is an important feature of aging and metabolic diseases; in diabetic kidney disease (DKD), renal NAD^+^ depletion arises from impaired synthesis and excessive consumption, which impairs NAD^+^-dependent enzymatic activity, exacerbates mitochondrial dysfunction, oxidative stress and renal inflammation, and thus drives renal cell injury and renal fibrosis to accelerate DKD progression (32). In DKD renal tubules, mitochondrial dysfunction leads to decreased NAD^+^/NADH ratio and impaired SIRT1/3 activity, promoting increased acetylation levels and mitochondrial dysfunction (29). Supplementation of NAD^+^ precursors (such as nicotinamide ribose) can restore the activity of SIRT family deacetylase of kidney proper cells (podocytes, mesangial cells, RTECs) in diabetes nephropathy (DKD), improve their mitochondrial energy metabolism and function, reduce oxidative stress, inflammation and fibrosis, and then alleviate DKD related kidney damage (22).

Succinate and fumarate, as TCA cycle intermediates, can competitively inhibit α-KG-dependent dioxygenases, leading to DNA and histone hypermethylation. Whether there is abnormal accumulation of succinate and fumarate in DKD and its effect on epigenetic modifications warrant further in-depth mechanistic exploration.

### 4.2 Epigenetic modifications feedback-regulate mitochondrial metabolism: a systematic framework

Epigenetic mechanisms can feedback to modulate mitochondrial metabolic status by regulating the expression of metabolism-related genes, forming a bidirectional regulatory circuit. While Section 4.1 focused on how metabolic intermediates influence epigenetic enzymes, this section systematically summarizes the reverse direction—how DNMTs, HDACs, sirtuins, and microRNAs (miRNAs) target key metabolic genes, particularly PGC-1α, CPT1A, and PPARα, thereby closing the feedback loop from epigenetics to metabolism. The feedback pathways are explicitly annotated in each subsection and integrated.

#### 4.2.1 DNMT-mediated DNA methylation suppresses metabolic gene expression

DNMTs, especially DNMT3B, are upregulated in DKD renal tubules under high-glucose conditions via TGF-β1/Smad3 signaling. DNMT3B directly binds to the promoter regions of PGC-1α, PPARα, and CPT1A, increasing CpG island methylation and recruiting methyl-CpG-binding proteins to suppress transcription. In DKD models, bisulfite sequencing confirms hypermethylation of the PGC-1α promoter (−450 to −120 bp relative to transcription start site (TSS)), correlating with reduced PGC-1α mRNA and protein levels, which impairs mitochondrial biogenesis and oxidative phosphorylation. Similarly, PPARα promoter methylation is elevated in proximal tubular cells from DKD patients, and treatment with 5-AzaC restores PPARα expression and downstream FAO gene expression. Hypermethylation of the CPT1A promoter further reduces fatty acid flux into mitochondria, promoting lipid accumulation. Notably, the methylation level of the TFAM (mitochondrial transcription factor A) promoter is also elevated in DKD, contributing to decreased mtDNA copy number and respiratory chain dysfunction. Importantly, DNMT3B upregulation itself is driven by oxidative stress and inflammatory signals, creating a pathological feed-forward loop: epigenetic silencing of metabolic genes → reduced NAD^+^ levels (as NAD^+^ synthesis depends on intact oxidative metabolism) → impaired SIRT1-dependent suppression of DNMT3B expression → sustained hypermethylation. This feedback pathway explains the persistence of metabolic reprogramming.

#### 4.2.2 HDAC/sirtuin-mediated deacetylation and transcriptional repression

HDACs remove acetyl groups from histone tails surrounding metabolic gene loci, compacting chromatin and repressing transcription. In DKD RTECs, class I HDACs (HDAC1/2) and class IIa HDAC4 are aberrantly recruited to the PGC-1α promoter via interaction with the transcriptional repressor FoxO1, reducing H3K9ac and H3K27ac marks. HDAC inhibitors (e.g., sodium butyrate, vorinostat) restore PGC-1α expression and ameliorate mitochondrial dysfunction. For PPARα and CPT1A, HDAC1-mediated deacetylation of histones at their promoters correlates with decreased expression in insulin-resistant states. Among sirtuins, SIRT1 and SIRT3 play dual roles: nuclear SIRT1 deacetylates PGC-1α to enhance its transcriptional activity, but under DKD conditions, NAD^+^ depletion (see Section 4.1) reduces SIRT1 activity, leading to hyperacetylated and inactive PGC-1α. SIRT3, the major mitochondrial deacetylase, regulates the acetylation status and activity of multiple metabolic enzymes including LCAD and SOD2. SIRT3 expression itself is regulated by epigenetic modifications (e.g., promoter methylation), and its reduced activity in DKD promotes mitochondrial protein hyperacetylation and dysfunction. This represents a key feedback node: impaired FAO → reduced acetyl-CoA → decreased histone acetylation at PPARα and CPT1A promoters → further transcriptional suppression of FAO genes → worsening lipid accumulation.

#### 4.2.3 miRNA-mediated post-transcriptional control of metabolic pathways

Multiple microRNAs upregulated in DKD directly target the 3′UTRs of PGC-1α, CPT1A, and PPARα. miR-29 is consistently elevated in DKD tubules and binds to the PGC-1α 3′UTR (seed region complementary to nucleotides 1572-1578), reducing PGC-1α translation without affecting mRNA stability. miR-33a and miR-33b, encoded within introns of sterol regulatory element-binding protein (SREBP) genes, directly target CPT1A and PPARα, and their expression is induced by high glucose via SREBP activation. Antagomir-mediated silencing of miR-33a in DKD mice restores CPT1A expression and FAO flux by ~60%. miR-21, one of the most consistently upregulated miRNAs in DKD, targets PPARα indirectly through sponging of its positive regulators, and also directly targets PTEN, leading to Akt activation, which in turn phosphorylates and inhibits FoxO1-mediated transcription of PPARα. This establishes a multilayered feedback loop: lipid accumulation → NF-κB activation → miR-21 transcription → PPARα suppression → further FAO impairment → more lipid accumulation. Additionally, lncRNA TUG1, downregulated in DKD via METTL3-mediated m6A reduction, regulates PGC-1α expression by sponging miR-29, representing another epigenetic layer of metabolic control.

#### 4.2.4 Integrated feedback pathways and the “metabolic memory” axis

The feedback nature of this regulatory axis is best exemplified by the NAD^+^-SIRT1-PGC-1α circuit. Epigenetic repression of PGC-1α (via DNMT3B hypermethylation or HDAC-mediated histone deacetylation) reduces mitochondrial biogenesis and oxidative phosphorylation, lowering the NAD^+^/NADH ratio. Since SIRT1 activity is exquisitely NAD^+^-dependent, reduced NAD^+^ further impairs SIRT1-mediated deacetylation and activation of residual PGC-1α, creating a vicious cycle of progressive metabolic decline. Similarly, miR-21-mediated suppression of PPARα reduces FAO, causing accumulation of long-chain fatty acyl-CoAs, which allosterically inhibit the ubiquitin-mediated degradation of DNMT3B, leading to DNMT3B protein stabilization and enhancing PPARα promoter methylation. These interconnected feedback loops explain the persistence of metabolic reprogramming even after glycemic normalization—the “metabolic memory” phenomenon (further discussed in Section 4.5, originally 4.4). Targeting these feedback pathways—through DNMT inhibitors (e.g., 5-AzaC, though with toxicity concerns), HDAC inhibitors (e.g., sodium butyrate), or miRNA antagomirs (e.g., anti-miR-21, anti-miR-33)—represents a promising therapeutic strategy to rebalance mitochondrial metabolism in DKD.

It is important to acknowledge that most of the evidence reviewed above derives from *in vitro* high-glucose conditions or rodent models, with limited validation in human DKD biopsies. Moreover, the strength of evidence varies considerably: the NAD^+^-SIRT1-PGC-1α loop is supported by multiple independent studies including pharmacological interventions and genetic models, whereas the proposed DNMT3B-SFRP5-Wnt-metabolism axis remains speculative due to the lack of direct metabolic readouts. Additionally, apparent contradictions exist—for instance, while HDAC inhibitors show renoprotection in some studies, global HDAC inhibition may disrupt normal metabolic homeostasis. These discrepancies highlight the need for cell-type-specific and stage-specific manipulations in future research.

All above scattered feedback pathways are integrated into our self-established bidirectional closed-loop regulatory model shown in **Figure 1**. Briefly, decreased NAD^+^ and acetyl-CoA derived from impaired FAO and OXPHOS reduce SIRT1/SIRT3 deacetylase activity and restrain histone acetylation, while insufficient α-KG triggers DNMT3B-mediated hypermethylation on promoters of PGC-1α, PPARα and CPT1A. Downregulated expression of these master metabolic transcription factors worsens mitochondrial structural and functional defects, which continuously disturbs the homeostasis of key metabolic intermediates and sustains abnormal epigenetic modification. Three hub molecules (DNMT3B, PGC-1α, SIRT3) serve as indispensable switching nodes connecting metabolism and epigenetics throughout the whole cyclic cascade. This visualized loop model distinguishes our review from previous descriptive summarization and constitutes our original theoretical framework.

### 4.3 Molecular mechanisms of bidirectional crosstalk: key node examples

The four representative molecular axes described below are critical constituent modules of our proposed integrated bidirectional regulatory loop model (**Figure 1**), rather than independent separated signaling pathways. Each axis interacts with one another via shared intermediate metabolites and core epigenetic enzymes to jointly maintain the pathological cyclic damage of RTECs in DKD.

#### 4.3.1 Bidirectional regulation of PGC-1α

PGC-1α is regulated by both epigenetic modifications and metabolic regulation. On the one hand, PGC-1α promoter methylation and histone acetylation status determine its expression level. On the other hand, PGC-1α acts as a transcriptional coactivator to promote mitochondrial biogenesis and the expression of FAO-related genes (33). SIRT1 mediated deacetylation of PGC-1α enhances its activity, while SIRT1 itself is regulated by NAD^+^ levels, forming a metabolic epigenetic regulatory loop. This regulatory link is embedded in the central cascade of our closed-loop model.

#### 4.3.2 SIRT3 mitochondrial metabolic axis

SIRT3 is the major deacetylase in mitochondria and regulates the activities of a variety of metabolic enzymes, including long-chain acyl-CoA dehydrogenase (LCAD) and SOD2 (34). SIRT3 expression is regulated by epigenetic modifications and transcription factors, and its activity is dependent on NAD^+^ levels. The decrease of NAD^+^ in DKD leads to impaired SIRT3 activity and promotes mitochondrial protein acetylation and dysfunction. As a core mitochondrial node in **Figure 1**, SIRT3 bridges NAD^+^ fluctuation and downstream epigenetic-metabolic changes.

#### 4.3.3 DNMT3B-SFRP5-Wnt axis

In DKD renal tubules, DNMT3B is upregulated and silences SFRP5 via promoter methylation, thereby activating the Wnt/β-catenin pathway and promoting renal fibrosis (19). Although Wnt signaling can regulate mitochondrial metabolism in other tissues, direct evidence linking this specific DNMT3B-SFRP5-Wnt axis to metabolic reprogramming in DKD RTEC injury is currently lacking. Thus, its role in the potential bidirectional crosstalk warrants further future experimental verification.

#### 4.3.4 miR-21/PPARα regulatory axis

miR-21 is upregulated in DKD, targeting PPARα to inhibit its expression, resulting in impaired FAO and lipid accumulation (35). Lipid accumulation in turn can affect the epigenetic modification through metabolic intermediates, forming a vicious cycle. The lipid accumulation-initiated vicious cycle is a key auxiliary branch of our whole-loop model.

### 4.4 Bidirectional regulation in the phenomenon of “metabolic memory”

“Metabolic memory” refers to the phenomenon whereby early glycemic exposure leads to persistent organ damage; this persists despite subsequent glycemic control exposure can still lead to sustained organ damage, which is particularly prominent in DKD patients. Epigenetic modifications are thought to be the molecular basis of metabolic memory (36). High glucose induced changes in epigenetic modifications can persist after glycemic control, maintaining abnormal gene expression patterns.

Mitochondrial metabolic reprogramming plays a key role in metabolic memory formation. High glucose induced mtROS can damage mitochondrial DNA and respiratory chain, leading to persistent mitochondrial dysfunction and metabolic abnormalities. At the same time, changes in mitochondrial metabolic intermediates can regulate the activity of epigenetic modifying enzymes and transduce metabolic signals into stable epigenetic marks. For example, the decrease of α-KG induced by high glucose can lead to the impairment of TET (ten-eleven translocation) activity and maintain the DNA hypermethylation state; Changes in the source of acetyl-CoA affect histone acetylation levels (37).

This bidirectional regulatory mechanism explains the persistence of metabolic memory: early metabolic stress induces mitochondrial dysfunction and epigenetic reprogramming, which mutually reinforce each other. Even if blood glucose returns to normal, this pathological vicious cycle persists.

**Figure 4:**
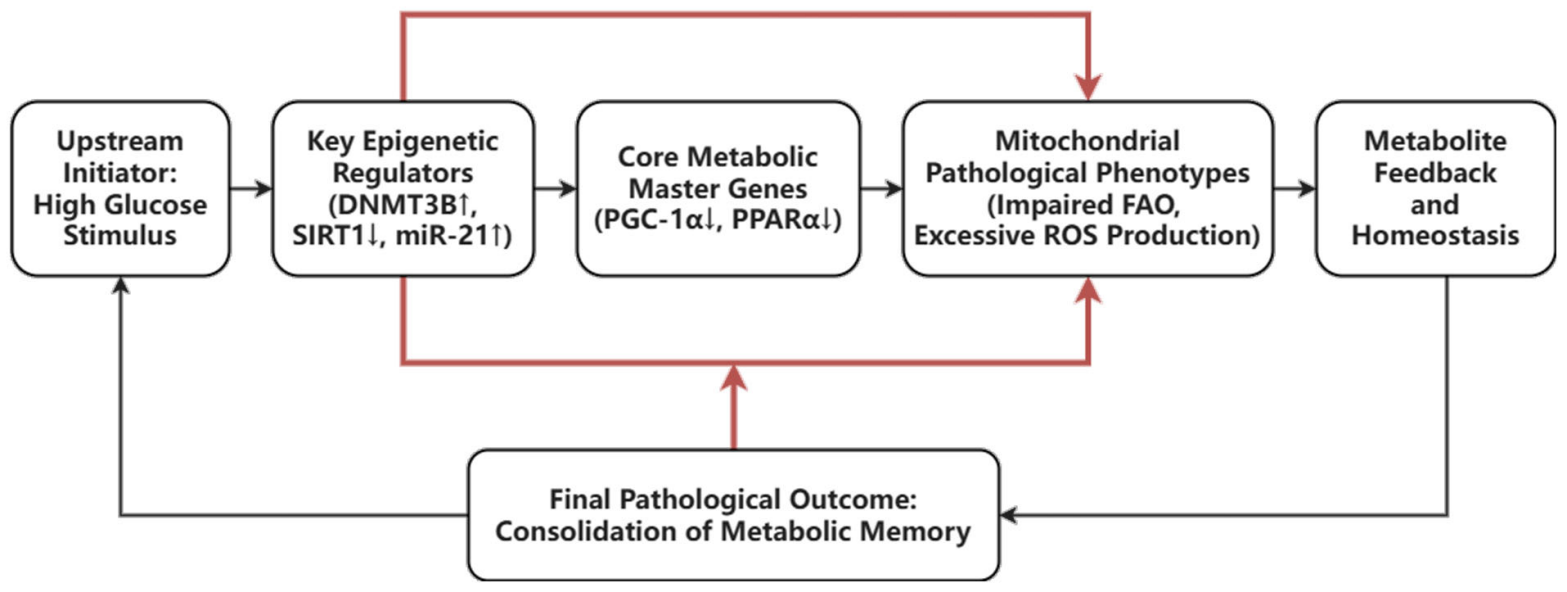
Bidirectional regulatory circuit between epigenetics and mitochondrial metabolism driving metabolic memory in DKD.

Based on the above systematic collation of bidirectional crosstalk between mitochondrial metabolic reprogramming and epigenetic modification as well as the molecular basis underlying metabolic memory, we have constructed an integrated cyclic regulatory schematic diagram to visually summarize this pathological closed-loop cascade, as shown in **Figure 4**.

## 5 Therapeutic prospects of targeting the bidirectional regulatory axis

### 5.1 Mitochondria-targeted therapeutic strategies

Mitochondria targeted antioxidant SS31 preserves mitochondrial cristae structure, reduces mtROS production, and restore mitochondrial function. In the DKD animal model, SS31 treatment significantly alleviated renal tubular injury, restored FAO function, and reduced lipid accumulation (16). Mechanistic studies revealed that the protective effect of SS31 was partially achieved by reversing metabolic reprogramming and restoring the mitochondrial epigenetic regulatory network.

MitoQ is another mitochondria-targeted antioxidant that exerts protective effects by scavenging mtROS (38). A phase IV clinical trial of MitoQ in patients with stage 3–4 chronic kidney disease (NCT02364648) has been completed, confirming MitoQ improves vascular endothelial function and arterial hemodynamics with good clinical tolerability. MitoQ may represent a promising strategy for managing vascular dysfunction in CKD, though further research in diabetic kidney disease is needed.

NAD^+^ supplementation strategies (such as nicotinamide ribose and nicotinamide mononucleotide) can restore SIRT1/3 activity in renal cells of diabetic kidney disease (DKD), improve mitochondrial oxidative phosphorylation and dynamic balance, inhibit renal inflammatory response mediated by mtDNA leakage, and alleviate podocyte damage, mesangial cell proliferation, and renal interstitial fibrosis, thereby alleviating renal pathological damage in DKD and improving renal function related indicators (22). In animal models of DKD, NAD^+^ supplementation alleviated kidney injury, indicating its translational potential.

Despite promising preclinical efficacy, mitochondrial-targeted agents face notable translational barriers. SS31 exhibits cell-type specificity concerns, with off-target effects in non-renal tissues at high doses, while its short half-life requires frequent administration and limits sustained renal accumulation. MitoQ, although well-tolerated in short-term trials, shows variable oral bioavailability and mitochondrial delivery heterogeneity across renal tubular segments, with long-term safety data in DKD populations still lacking. For NAD^+^ supplementation, precursors such as nicotinamide riboside (NR) and nicotinamide mononucleotide (NMN) demonstrate dose-dependent toxicity including hepatotoxicity and gastrointestinal disturbance at high doses; moreover, renal-specific delivery remains a critical challenge, as systemic supplementation alters NAD^+^ homeostasis in multiple organs, potentially disrupting physiological metabolism. Current clinical trials (e.g., NCT02364648) are limited by small sample sizes, short follow-up durations, and lack of renal tubular-specific outcome measures, hindering definitive conclusions on efficacy and safety in DKD patients.

### 5.2 Therapeutic strategies of epigenetic modification

5-AzaC, a DNA methyltransferase inhibitor, attenuates renal fibrosis in an animal model of DKD (19). However, as a broad-spectrum methylation inhibitor, 5-AzaC can cause genome-wide hypomethylation, induce chromosomal instability, hematologic toxicity, and abnormal activation of transposable elements, leading to potential genotoxicity and off-target gene expression disorders, thus restricting its clinical use (39). It is more promising to develop strategies that include pharmacological inhibition with DNMT3B-selective small molecules (e.g., Nanaomycin A), CRISPR-dCas9-DNMT3B/TET targeted epigenome editing for locus-specific methylation modulation, and RNA interference/shRNA-mediated genetic silencing of DNMT3B to avoid global hypomethylation and off-target toxicity (40).

HDAC inhibitors (such as vorinostat and trichostatin A) showed protective effects in DKD models, restoring PGC-1α expression and improving mitochondrial function (41). However, the off-target effects and toxicity of HDAC inhibitors require careful evaluation. Selective HDAC inhibitors or SIRT activators may be more advantageous.

Therapeutic strategies targeting noncoding RNAs are also in development. Key advances include APOC3-targeted antisense oligonucleotides (ASOs) (preclinically mitigating DKD and atherosclerosis in murine models) and GalNAc (N-acetyl-D-galactosamine)-conjugated siRNAs (small interfering RNAs) (e.g., plozasiran, RN0361) in advanced clinical trials for hypertriglyceridemia, alongside miRNA/mimic and lncRNA modulation strategies under preclinical evaluation for APOC3-linked metabolic renal disease (42). ASOs and miRNA mimics/inhibitors have shown potential in preclinical models (42).

Epigenetic therapies encounter substantial specificity, toxicity, and delivery limitations in DKD translation. Broad-spectrum DNMT inhibitors such as 5-azacytidine induce global DNA hypomethylation, leading to chromosomal instability, hematological toxicity, and aberrant activation of transposable elements; locus-specific delivery remains unachieved with current systemic formulations, causing widespread off-target gene dysregulation. HDAC inhibitors (e.g., vorinostat, trichostatin A) show class-wide toxicity including cardiotoxicity and metabolic disturbances, with poor renal tubular selectivity limiting their therapeutic index. Non-coding RNA-based strategies face major delivery hurdles: ASOs and siRNAs suffer from rapid renal clearance, nuclease degradation, and inefficient cellular uptake in RTECs; while GalNAc-conjugated modifications improve liver targeting, renal-specific delivery systems are still in preclinical development. Additionally, most ncRNA targets exhibit context-dependent expression across DKD stages, and off-target effects on non-renal cells remain poorly characterized, restricting clinical trial design and patient selection.

### 5.3 Combination therapy strategies targeting bidirectional regulatory axis

Given the complexity of the bidirectional crosstalk network, it may be difficult to fully halt DKD progression by targeting mitochondria or epigenetics alone. The combination therapy strategy is worth exploring. For example, combination of mitochondrial antioxidants (e.g., mitoQ, apigenin, resveratrol) and NAD^+^ precursors (NR/NMN) synergistically restores SIRT1/3 deacetylase activity, reduces mitochondrial ROS, normalizes NAD^+/^NADH ratio, and improves renal mitochondrial function in diabetic kidney disease models more effectively than single agents (43); DNA methyltransferase inhibitors combined with PPARα agonists may improve metabolic function through different mechanisms (44).

In addition, regulating the level of metabolic intermediates (such as α-KG and β-hydroxybutyrate supplementation) may play a therapeutic role by affecting the activity of epigenetic modifying enzymes. The safety and effectiveness of these strategies still require clinical validation.

Combination therapies targeting the bidirectional regulatory axis offer synergistic potential but face compounded translational challenges. Co-administration of mitochondrial antioxidants and NAD^+^ precursors may exacerbate systemic toxicity due to overlapping off-target pathways, while pharmacokinetic interactions (e.g., altered drug metabolism) require rigorous dose-adjustment studies. The combination of epigenetic modifiers and metabolic regulators further complicates specificity control, as concurrent epigenetic and metabolic perturbations may induce unforeseen gene expression changes and cellular stress. Renal-specific co-delivery systems (e.g., liposomal nanoparticles, tubular-targeted peptides) are still under preclinical validation, with scalability and manufacturing challenges delaying clinical translation. Furthermore, DKD heterogeneity across patients (e.g., varying degrees of tubular injury, metabolic status) necessitates personalized therapy design, but current biomarkers for patient stratification are insufficiently validated, limiting the generalizability of combination therapy efficacy in clinical settings.

### 5.4 Clinical translation challenges and potential solutions

Despite the promising therapeutic strategies discussed above, several major obstacles must be overcome before they can be translated into clinical practice for DKD. First, broad-spectrum epigenetic drugs such as the DNA methyltransferase inhibitor 5-azacytidine (5−AzaC) carry significant extrarenal toxicity, most notably bone marrow suppression, which has limited their use to hematological malignancies. Even at low doses, systemic administration of 5−AzaC can cause myelosuppression, gastrointestinal adverse effects, and potential genotoxicity due to global hypomethylation. Second, kidney-specific delivery remains a formidable challenge. Many small-molecule inhibitors (e.g., DNMT3B inhibitors, HDAC inhibitors) and metabolic intermediates (e.g., NAD^+^ precursors) lack intrinsic renal tropism. After systemic administration, they may not achieve sufficient concentrations in proximal tubular epithelial cells—the primary site of DKD pathology—while exposing other organs to potentially toxic levels. For instance, achieving a therapeutically effective intrarenal concentration of a DNMT inhibitor without causing systemic hypomethylation is extremely difficult. Third, metabolic intermediates such as α-KG and NAD^+^ are chemically unstable in circulation and have short half-lives. Moreover, because both intermediates and epigenetic modifiers control fundamental cellular processes including proliferation and differentiation, their long-term systemic modulation raises concerns about tumorigenesis. Aberrant activation of TET-mediated DNA demethylation or SIRT-dependent deacetylation could potentially promote oncogenic transformation, especially in patients with diabetes who already have an altered epigenetic landscape.

To address these challenges, several innovative solutions are being explored. For extrarenal toxicity, isoform-selective or locus-specific epigenetic modulators are urgently needed. For example, selective small-molecule inhibitors of DNMT3B (e.g., nanaomycin A) or CRISPR-dCas9-based epigenome editing tools could allow precise methylation modification at the SFRP5 promoter without affecting global DNA methylation. For kidney-targeted delivery, nanoparticle-based carriers (e.g., polymeric or lipid nanoparticles) conjugated with renal tubular-targeting ligands (e.g., megalin/cubilin ligands or peptide motifs such as LyP-1 and KH) can enhance drug accumulation in proximal tubules while reducing systemic exposure. Likewise, prodrug strategies that are activated specifically by renal enzymes (e.g., γ-glutamyl transferase or aminopeptidase N) offer another avenue for tubule-selective release. For improving the *in vivo* stability of metabolic intermediates, chemical modification (e.g., nicotinamide riboside and mononucleotide for NAD^+^, cell-permeable ester forms of α-KG) or encapsulation in protective delivery vehicles can prolong their half-life. The long-term safety of these intermediates should be rigorously evaluated in large-animal models with extended follow-up to monitor for any neoplastic potential, and combination with low-dose anti-inflammatory agents may help mitigate off-target proliferative risks. Ultimately, patient stratification based on baseline methylation or NAD^+^ profiles may help identify those most likely to benefit while minimizing hazards. These translational hurdles must be systematically addressed before the bidirectional regulatory axis can be successfully exploited for DKD therapy.

## 6 Conclusion and prospect

This paper expounds the bidirectional regulatory relationship between mitochondrial metabolic reprogramming and epigenetic modification in renal tubular injury in DKD. The core points can be summarized as follows:

### 6.1 Critical appraisal of current evidence

This review recognizes several conceptual and evidential limitations. First, the bidirectional regulation framework, while heuristic, largely derives from correlative studies; direct causal evidence—such as tissue-specific epigenetic enzyme knockout reversing metabolic phenotypes-remains scarce in DKD. Second, most data come from proximal tubule models, leaving distal nephron segments and collecting duct cells largely unexplored. Third, the field has not yet established a temporal hierarchy: which event (metabolic reprogramming or epigenetic alteration) occurs first in human DKD progression remains unknown. Fourth, translational potential is hampered by the lack of renal-selective delivery systems and concerns over off-target toxicity of epigenetic drugs.

### 6.2 Future research directions

To address this critical limitation, future studies should integrate advanced technologies to resolve segment-specific regulatory mechanisms. Single-cell metabolomics can profile metabolic pathway activities at individual cell-type resolution. Spatial transcriptomics can map gene expression and epigenetic signatures *in situ* along the nephron axis. Laser capture microdissection (LCM) of distinct nephron segments can provide isolated, segment-specific material for targeted multi-omics analysis. These approaches will help clarify whether the bidirectional crosstalk between mitochondrial metabolism and epigenetic modifications operates through segment-specific molecular logic and support the development of segment-targeted therapeutic strategies for DKD.

First, mitochondrial metabolic reprogramming is the central event in renal tubular injury in DKD, which is manifested by impaired oxidative phosphorylation, enhanced glycolysis, impaired FAO and lipid accumulation. These metabolic changes are regulated by multiple signaling networks such as AMPK, PGC-1α, HIF-1α, etc.

Second, epigenetic modifications (DNA methylation, histone modification, non coding RNA) play a key regulatory role in renal tubular injury in DKD, and participate in the pathogenesis by affecting the expression of metabolism, inflammation and fibrosis related genes.

Third, there is a complex bidirectional regulation between metabolic reprogramming and epigenetic modification: metabolic intermediates (acetyl-CoA, α-KG, NAD^+^), as substrates or cofactors of epigenetic modifying enzymes, directly regulate the epigenetic modification state. Epigenetic mechanisms influence mitochondrial function by regulating metabolism-related gene expression. This bidirectional regulatory network constitutes the molecular basis of “metabolic memory” of DKD.

Fourth, the therapeutic strategies targeting the bidirectional regulatory axis (mitochondrial targeted drugs, epigenetic modifiers, and metabolic intermediate supplementation) show promising therapeutic potential, and combination therapy may exert synergistic effects.

However, this field still faces many challenges, and there are several promising research directions. First, this review focuses predominantly on proximal tubular epithelial cells, but cell specificity remains underexplored; how bidirectional regulation differs across distal tubules, collecting duct cells, glomerular-tubular crosstalk, and immune cell contributions requires further investigation (44). Second, as DKD progresses, metabolic reprogramming and epigenetic modification patterns may change dynamically, and longitudinal studies are helpful to delineate this dynamic process. Third, the role of mitochondrial–nuclear signaling transduction (such as mitochondrial unfolded protein response and mitochondrial DNA release) in bidirectional regulation is worth further discussion. Fourth, single-cell multi-omics technology is expected to resolve metabolic epigenetic regulatory networks at single-cell resolution.

In conclusion, the bidirectional regulation of mitochondrial metabolic reprogramming and epigenetic modification provides a novel perspective for understanding the mechanism of renal tubular injury in DKD, and also points out the direction for developing new therapeutic strategies. Future studies need to further clarify the molecular details of this regulatory network and accelerate its clinical translation.
